# Are we on the cusp of a fourth research paradigm? Predicting the future for a new approach to methods-use in medical and health services research

**DOI:** 10.1186/s12874-018-0597-4

**Published:** 2018-11-14

**Authors:** Frances Rapport, Jeffrey Braithwaite

**Affiliations:** Centre for Healthcare Resilience and Implementation Science, Australian Institute for Health Innovation, Macquarie University, Level 6, 75 Talavera Road, North Ryde, NSW 2109 Australia

**Keywords:** *Fourth research paradigm*, *Third research paradigm*, Mobile methods, Qualitative research, Methodological development, Complexity, The future of healthcare

## Abstract

**Background:**

The dominant medical and health research paradigm continues to be quantitative. While the authors sense a sea-change in opinion about mixed-method research, underpinned by two decades of highly-cited publications in medical journals, much of the medical literature still widely favours the Randomised Control Trial.

**Main body:**

This debate article examines whether it is the beginning of the end of the dominant quantitative paradigm and the interest this holds for researchers and clinicians at the forefront of care delivery. It examines the *Third Research Paradigm,* signifying the importance of mixed-methods, and discusses the power of the patient voice and person-focused research activity. The authors discern the coming of age of a *Fourth Research Paradigm* integrating mixed-methods with data collected ‘on the hoof’. Within this new paradigm, the article explores the power of available, real time, and emergent data – from smart phones, wearable devices, and social media, as well as more creative approaches to data collection. The *Fourth Research Paradigm* will require the support of multi-disciplinary teams, moving through the world alongside their research subjects. The impact of a *Fourth Research Paradigm* on the health researcher is assessed, as the researcher’s gaze moves away from considerations of methodological superiority to re-considerations of their role in the brave new world of research multiplicity.

**Conclusion:**

The *Fourth Research Paradigm* offers extensive opportunities to tell more complete research stories in real-time settings. It concentrates on contextual notions of everyday happenings within the ever-changing world of healthcare delivery. There will be challenges ahead, not least the management of large, complex datasets and adaptive study designs. But rigorous planning will enable unique insights into the relationships played out in the world of the patient and healthcare provider. Better care and new delivery models are likely to result, but how this will manifest is not yet clear.

## The role of qualitative methods in the future of health services research

Between 1993 and 2000, an influential series of papers on the role of qualitative methods in medical and health services research was warmly received (See for example [1–3]). Following publication, the series sparked extensive and ongoing interest in new opportunities for mainstream medical research to incorporate qualitative methods as a matter of course. Years later, however, a group of 76 senior academics from 11 countries took issue with the *British Medical Journal* for rejecting qualitative research contributions too readily, and for instead privileging quantitative studies, particularly randomised controlled trials (RCTs) [4]. The 76 advocates showed that the citations for the most influential qualitative research papers were much higher than the three top-nominated RCTs that the BMJ cited, and were amongst the most influential papers to have made their way into online publishing in over 20 years.

In the intervening period, from 1993 to now, much has happened in the field of qualitative research. In this paper we aim to bring readers up to date with what qualitative research has contributed and what it has yet to contribute, in order to indicate what the future holds for it. We ask: is there a new paradigm emerging, one which conjoins qualitative and quantitative approaches with new, more flexible, real-time data?

## The beginning of the end of a dominant paradigm?

The dominant paradigm in health and medical research remains steeped in positivistic thought. Despite debate on the topic [5], the gold standard (in terms of effectiveness studies with measurable primary and secondary outcomes that claim internal validity) is still widely accepted as the RCT. However, it is worth noting that any study which is predicated on summarising its results into a series of numbers is, by any definition, highly subjective, with personal judgements made about what is valuable and worthy of measure, how measurement should be undertaken, and what should and should not be reported – and even, what the numbers mean, and how they are interpreted by different shareholder groups. Nevertheless, this type of work still claims objectivity in terms of measurement and outcome. A number, that makes an objective claim as a statement of fact that can be proved, such as those found in trials, depicting the effect of, for example, services on quality of life [6], measuring the effect of a drug on a patient’s response to chronic symptoms [7], or symptom change resulting from drug adherence [8], are, like all other research studies, simply abstractions of reality. Meanwhile, the case could be made that undertaking a richly detailed social observation study examining the behaviour of clinicians and patients directly by working alongside them for an intensive period of time, in situ, using well-known ethnographic techniques, is a truer reality. Which, then, is the gold standard? To examine their experiences, note their opinions, and watch and learn what participants are going through reveals so much about health care in the real world. Thus, both trials and ethnographic studies examine different aspects of medical procedures and patient care, each focusing on trying to understand the human condition in the healthcare context from a different angle and each ‘constructing’ data that is, inevitably, subjective.

Health care professionals appear to be starting to appreciate the scope of qualitative methods for examining their complex worlds, at both strategic and practical levels. This is evidenced, not only by greater Government funding for qualitative trials’ work (see for example the Welsh Assembly Government’s recent funding for the initiative: the ‘Qualitative Enquiry Supporting Trials (QUEST)’ unit in Wales (2013-ongoing), but also the steady growth in the number of published trials in medical journals that include a qualitative arm (see for example: [9, 10]. Health care professionals are not only recognising methods within the traditional qualitative researcher’s portfolio, but also new methods that are becoming *de rigueur* for qualitative methodologists working within clinical and non-clinical trials, including visual taxonomies [11, 12], bio-photographic techniques [13], and summative and schema analyses [14–16]. It is now much more common to see qualitative methods applied to drug, device and therapeutics trials, while qualitative methods have their own set of Standard Operating Procedures (SOPs) [17] and can hold their own in the face of complex, multi-facetted study designs. While imagination and creativity are vitally important aspects of qualitative research development, this does not rule out the opportunity to standardize operational procedures and offer greater structure as a result to certain kinds of studies such as clinical trials and cohort studies. Indeed, SOPs are invaluable for recognising which interventions translate across settings and which do not, and how to develop new theories of organisational change relevant to patient safety and quality studies [18–20]. They can even tease out what happens when implementation of an intervention goes wrong, satisfying our desire to reveal evidence-deficits and methodological flaws [21–23].

## More than a sea change?

Reflecting on other’s research, and conducting our own in both qualitative and quantitative modes for several decades, we believe that a sea-change is taking place, where the causal explanation and standardized outcome measurement is no longer sufficient. But more than this, leading healthcare researchers are now seeking out a more nuanced, fine-grained understanding of health care performance than causal explanations can offer [24, 25], envisaging more comprehensive, triangulated designs, greater scrutiny of clinical and system-wide data, and new ways of interpreting datasets.

To set this sea-change in context, we briefly outline the paradigm shifts that have taken place in health services and medical research to date. A paradigm shift is a Kuhnian notion indicating a radical theory change to the commonly held beliefs and agreements scientists share at a point in time. In complexity science terms, this represents a phase transition to a new order [26].

To begin with there was the *First Research Paradigm*, with its positivistic notions of the supremacy of numbers based on scientific objectivity [27]. This was followed by the *Second Research Paradigm,* which argued for the use of qualitative methods, which endorsed researcher subjectivity, and the “superiority of constructivism, idealism, relativism, humanism, hermeneutics, and, sometimes, postmodernism” [28]. Over time, leading thinkers began to see that the two need not be the province of completely estranged groups (“scientists” and “social scientists”) but there were benefits in collaborating across the divide. The *Third Research Paradigm,* argued for methodological pluralism, the use of multiple methods in combination, and the conscious coupling of qualitative and quantitative data, through data triangulation and inter-textual analysis [29]. The *Third Research Paradigm* found convergence a more potent message than exclusivity, while notions of a dominant, 'hard' statistical paradigm and a ‘soft’ qualitative option were increasingly rejected [16]. The *Third Research Paradigm* meant that fewer researchers dismissed qualitative methods as secondary to quantitative methods, while many welcomed their scope for bringing a depth and breadth to 'hard' data during collection, analysis and reporting stages.

The work of the *Third Research Paradigm,* accompanied by the triangulation of datasets and data sources, personalised many of the benefits of multi-methodology, strengthening a study’s validity and the veracity of its working techniques [30]. In their various guises, inter-methods triangulation was written into new research studies. Whilst triangulation of different datasets is by no means easy, especially when it accounts for data collected according to different methods from a range of perspectives, if it can be applied systematically it can have significant benefits in aiding understanding, and can have a sustained effect on service improvement and the successful implementation of study outcomes [31].

## Climbing out of the hole, and going mobile

In 2004, Gareth Williams [32] described the way we examine (and to a degree we still continue to do so) research explanations and understandings according to methodological “bunkers”. This, he said, was a way of seeing the world that was: “impoverished, desiccated and confined” (xvii), and which resulted from a desire to:

*“… dig a hole, stick the name of a discipline or a method on it, get into it, and talk only to those who want to get into the hole with us; [who] are only allowed in once they have learned the methodological rules.”* (xvii). [32]

Since then progress has been made to get out of the hole and look around, but we are not in the clear yet, running free. We need to create a climate where researchers can be less fearful of: “using [their] imaginations” (xvii) to explain their perceptions of the world in which we live.

In such a climate, rather than playing: “according to the rules” (Williams 2004 xix) [32], researchers must let their imaginations roam. The resurgence in the philosophy, sociology and psychology of language known as the linguistic turn [33], meant that the spoken word gained a newfound popularity. Researchers should also embrace literary experimentation, and as they ‘show’ and ‘tell’ participant stories, be allowed to search their methodological repertoire for still and moving images to support biography [34, 35]. Lamprell and Braithwaite, for example, have shown how Joseph Campbell’s “The hero with a thousand faces” [36] applies in many areas of healthcare, including the patient’s journey across time [37].

## The fourth research paradigm

We have noticed, as paradigms shift and change and as new paradigms are introduced and accepted that there is a greater reliance on different forms of knowledge and different types of engagement with the world, through new epistemological and theoretical enquiries, than ever before (see Table 1 for a summary of four research paradigms). As a result, researchers will need to not only rely on tightly gathered statistics, or on the spoken and written word, but also on different data types, such as visual and performative data. It is only through a multiplicity of data sources that a more comprehensive rendering of the world under enquiry can be realised. Researchers will need even more to embrace creativity, and consider the interdisciplinary resources at their disposal, with different forms of scholarship needed to exploit these new resources. (See for example current research projects relying on the intersection between the spoken word and ethnographic poetic representation [38], or the cross-fertilisation of ideas from anthropologists, human geographers and health services researchers [39]. As a result, the authors believe we are positioning to welcome the coming-of-age of the *Fourth Research Paradigm.*

**Table 1:** The Paradigms of Research: Principal Features

	First paradigm	Second paradigm	Third paradigm	Fourth paradigm
Epistemology	Scientific, Evidence-based	Social Scientific, Qualitative	Multi-Method, Pragmatism	Fluid, Creative, Exploratory
Data type	Statistical, Precise, Explanatory	Linguistic, Descriptive, Interpretive	Combined (Statistical and Linguistic)	Mobile, Shared, Emergent
Research-subject relationship	Objective, Standardized	Subjective, Personalized	Pluralist, Versatile	Democratic, Equalizing, Adaptive
Researcher position	Inquisitor, Realist	Observer, Interpreter	Witness, Aggregator	Partner, Listener
Methodology	Randomised Controlled Trial, Before and After Study, Time Series, Interrupted Time Series, Cohort Study, Systematic Review	Ethnography, Case Study, Phenomenology, Grounded Theory, Meta-Ethnography, Meta-Narrative	Mixed-Methods (Verbal, Visual and Numerical), Meta-Synthesis	Performative, Science-Social Science, Ethnographic, Poetic Representation
Methods	Scientific Measurements and Computational Techniques	Focus Groups, Interviews, Open-ended Questionnaires/Proformas, Observations, Participant Observations	Quantitative and Qualitative Data that can be Synthesized	Multiple methods from other paradigms, plus Technological Data (Smartphones, Apps), Social Media Data, Performance, Biographies and Photographs, Everyday Objects, Obscure Phenomena, Auto-biography
Data Characteristics	Set, Precise, Population-based, Explanatory	Interrogative, In-depth, Rich, Patterned	Dichotomous, Situational, Pragmatic, Triangulated	Nuanced, Ambiguous, Complex, Anomalous, Flexible

While the *Third Research Paradigm* is far from fully established, the *Fourth Research Paradigm* not only brings quantitative and qualitative methods together more coherently (from a more pressing need to appropriate the right mix of methods in any given research situation for useful outcomes), but seeks to add available or emergent data from a wide range of sources needed to render a more complete picture of the world, including those reliant on big data gathered outside of research and data from technological devices and social media sources. The *Fourth Research Paradigm,* in effect, draws on all sources while extending ethnographic methods to bring into its own the mobile method. Facilitating the mobile method, researchers would purposefully conduct their research in real time ‘on the hoof’, moving through the world alongside their research subjects, gathering or exploiting data from as many available sources as necessary, thereby to ensure realistic changes to practice are noted and visualised [40]. As a result, there will be even more growth of data types than we have seen to date. If this occurs in the way we are imagining it, researchers will welcome a next generation of creative insights, mixed-disciplinary expertise and fluid movement through research spaces alongside healthcare professionals, patients and other stakeholders. The *Fourth Research Paradigm* thus heralds a seismic shift in the world of health services and medical research, while researchers will move away from their siloed balkanisation, and shift their gaze from considerations of methodological superiority and inferiority to considerations of their role as research bricoleurs. This can come about because the *Fourth Research Paradigm* emphasises the need to concentrate, not so much on what makes the paradigmatic camps different and separate, but rather what the right mix of data can offer to all involved. Going further, researchers will recognise the importance of working ‘alongside’ their research subjects, translating as they go, while research subjects will recognise the value of having a stake in the real-time collection of data and in-time access to research findings to bring about change that suits their needs through greater clarity of shared intentions and clear, implementable outputs. Thus, rather than working ‘on’ subjects, methodological supremacy in this scenario could transcend into methodological inclusivity.

In the *Fourth Research Paradigm* researchers will appropriate the skills that are necessary to appreciate the actions, reactions and interactions of others, to act sensitively in front of others, to take into direct account coal-face behaviours and concerns, to listen more intently to what others have to say, and to co-design, co-develop and co-implement studies and their findings. Indeed, the *Fourth Research Paradigm*, with its reliance on a range of stimuli, such as technologically-driven data forms, will lead to new research design ideas, and a new integrity to the ‘space and place’ of the research subject and researchers will move ‘with and alongside’ research subjects. This differs substantially from interventional research and the ethnographic case study in both intent and conduct. While interventional researchers stimulate change in the active group and compare these outcomes to the control group and ethnographers concentrate on exposing cultural and social relationships, those steeped in the *Fourth Research Paradigm* will have a range of opportunities to conduct co-implementation with those researched, and include informal conversations, pathway maps, technological data, performative and creative writing data, personal biographies, and data in visual formats. This means working shoulder-to-shoulder with those providing and needing care, and exposing clearer insights into the active and ever-changing worlds of the provider and receiver of services. This will lead to a greater understanding of health systems, processes, and treatments, and greater clarity around the challenges facing healthcare providers and patients as they interact with each other within complex systems. As a result, researchers will spend more quality time examining what service providers actually do, and what care patients actually want, paving the way for greater attention being paid to ‘work-as-done’ rather than ‘work-as-imagined’ [41]. Thus, the paradigm shift will ensure research reporting is more accountable and rigorous, and service providers’ and patients’ experiences are more fully integrated. This will result in better-quality data, and instead of cross-sectional research which essentially realises brief, one-off soundbites from time-limited interviews or focus groups, greater long-term involvement will provide more comprehensive, nuanced data, indicating what is actually happening in the real, messy world of healthcare. In essence, this would amount to more radical research, an emphasis on teamwork and a democratisation of research. Both service providers and patients will have a more equal partnership, and have a voice in data collection, associated research transactions, and implementation, than was previously the case.

Whilst the demand for a *Fourth Research Paradigm* comes from the shortcomings of prior research paradigms, the paradigm-shift offers scope for changes to our healthcare systems and services that is far-reaching and impactful. The introduction of greater creativity and flexibility in data capture could bring about new models of data ownership, including greater attention being paid to every stakeholder’s interests. It will also mean that research will be less hierarchical and more heterarchical: not so elite, and more egalitarian. Interactions will be more horizontal and less vertical in arrangement. This is not to suggest that researchers will be spending their lives following clinicians around a hospital unit or ward, or that service providers will have to spend greater time in research laboratories. Rather, it will mean that data that is collected takes account of real-time behaviours and all stakeholders’ perspectives, that far more thought goes into the set-up of data collection and the techniques necessary to gather it collaboratively, and that more creativity is welcomed, to enable techniques to be put in place that allow for the data that is needed to answer the questions being asked. It will also ensure that more evidence can be offered about work-as-done rather than work-as-imagined. As a result, the reporting of data findings will likely have greater accuracy, depth and impact.

In effect, the *Fourth Research Paradigm* will not only fulfil a need for researchers to ensure their work is reality-based and meaningful, but that healthcare practitioners and patients can incorporate a model for the real world they inhabit – to show and tell their own experience and the challenges they face. They become, as a result, much more active participants in the research, with a greater symbiosis to that relationship, while rendering, more cogently, their daily lives, reducing the opportunity for researcher presumption.

Despite these multiple strengths, the *Fourth Research Paradigm* will challenge researchers immensely. For while enabling ‘real world’ insights into the relationships played out in the world of the healthcare provider, recording information in real-time raises notions of consent to a new level of complexity. Undertaking data collection on the hoof, the usual preparatory work that would indicate who counts as a study subject, who should be involved in data collection, who owns the collection, who is responsible for implementation, translation and change, and even what should stand for data, will not be possible. As the world of the researcher, healthcare professional and the patient expand and contract, with multiple system changes affecting service delivery, the collaborators will need to work out how to accommodate change in an environment where they are never entirely sure who they might meet, what will happen when they do so, and what real-time data will be available to them as they move through the world.

Furthermore, multi-constituency interactions mean that anonymity and confidentiality, at the researcher’s first point of contact, cannot always be assured. This is similar to the issues of anonymity and confidentiality in ethnographic settings, where others, who have not themselves consented to participate may enter into the frame of the recording of a real-life interaction. But in this case this can also include virtual interactions, which opens a whole area of new complexity, where people’s identifiers can be subsequently revealed in unexpected ways, Nevertheless, in medical and healthcare contexts, mobile methods offer realistic insights into the everyday experiences and interactions of patients and professionals, overtaking the stultified interview or the staged randomised trial in their reality-check. Mobile methods will also come to depend, more and more, on new technologies in healthcare settings [42] as researchers use data from Apps and tablets as sources of data, exploit social media, borrow from Twitter and Instagram, and secure information from medical devices. This means that the *Fourth Research Paradigm* will be at the forefront of technological breakthroughs in medicine, and access information from AI, genomics, personal biographies and electronic patient medical records. Indeed, we foresee the future of research as inscribing our response to the world through a greater use of technologically-derived data, as people look for new and better ways to map patient pathways, tell human stories through the objects that surround them, and streamline services.

## The next frontier

If and when the *Fourth Research Paradigm* truly takes a hold, researchers will be expected to handle ever-larger datasets from fragmented sources, assess the needs of ever-expanding populations and select research team-front line collaborators who are dependent on a growing number of interdisciplinary configurations. Outputs will need to be responsive to many datasets, and there will be the need for greater flexibility built into study designs so patients, the public and stakeholders have their say in data gathering methods and the delivery of health services. It is not at all clear how data from different sources can be weighted and fashioned into a coherent rendering. Amongst the greatest challenges are those that are methodological – how to ensure that the research designs we chose are rigorous and able to withstand the test of time, and how to develop theories that make sense of complex findings.

To manage these challenges, we will need to reach out to many others for assistance, and borrow techniques across fields. Whilst the authors cannot predict what will happen as researchers move towards the fourth paradigm, we sense that researchers are already embracing an ever-expanding palette of ideas, approaches, resources and affects to answer their complex research questions. In part, this expansiveness and inclusivity stems from the need to define new theoretical and epistemological connections between data in the face of rapid service adaptation. In part, this is the result of a backlash against the once firmly held, purist belief in the supremacy of one paradigm over another (as we have seen with the emergence of more mixed method approaches to data collection, analysis and interpretation). We believe we will be moving towards a greater sharing of methodological know-how, to help others research and theorise, while the next frontier will no doubt be to captivate the interest, excitement and ultimately the involvement of new stakeholders. With these new ways of working, and by balancing giving and taking, we predict a movement towards a greater understanding of the sweet, textured individuality of the human condition.
